# “A second birthday”? Experiences of persons with multiple sclerosis treated with autologous hematopoietic stem cell transplantation—a qualitative interview study

**DOI:** 10.3389/fneur.2024.1384551

**Published:** 2024-05-01

**Authors:** Timo Volz, Anna Sippel, Felix Fischbach, Johanna Richter, Alice Grizzel Willison, Vivien Häußler, Christoph Heesen

**Affiliations:** ^1^Institute of Neuroimmunology and Multiple Sclerosis (INIMS), University Medical Center Hamburg-Eppendorf (UKE), Hamburg, Germany; ^2^Department of Neurology, University Medical Center Hamburg-Eppendorf (UKE), Hamburg, Germany; ^3^Department of Stem Cell Transplantation, University Medical Center Hamburg-Eppendorf (UKE), Hamburg, Germany; ^4^Department of Neurology, Medical Faculty University Hospital Düsseldorf, Heinrich Heine University, Düsseldorf, Germany

**Keywords:** multiple sclerosis, hematopoietic stem cell transplantation, patient experiences, decision making, qualitative study, thematic analysis

## Abstract

**Introduction and objective:**

Autologous hematopoietic stem cell transplantation (aHSCT) is a promising treatment option for persons with multiple sclerosis (pwMS). Patients undergoing aHSCT face unique challenges in all aspects of life. In this study, we explored the lived experiences of pwMS undergoing aHSCT.

**Methods:**

Semi-structured interviews of 12 pwMS treated with aHSCT were conducted using a maximum variation sampling strategy. Interviews were transcribed verbatim and analyzed thematically using inductive and deductive categories.

**Results:**

Three major themes were identified: (1) preparing for aHSCT, (2) experiencing the procedure, and (3) post-treatment time. A difficult decision-making process, organizational effort, and funding difficulties characterized the preparation for transplantation. AHSCT was seen as a life-changing event accompanied by both psychological and physical stress, with an associated feeling of regaining control. The transplantation had a lasting positive effect on the lives of the interviewed pwMS. However, the early post-treatment time was characterized by successes and failures alike. Particularly the independently organized medical aftercare was perceived as challenging. Retrospective revaluation has led most pwMS to wish for earlier information provision about the treatment option of aHSCT during their treatment history.

**Conclusion:**

AHSCT had a clear impact on patients’ physical and psycho-social health, influencing their perception of life and its quality. Assessing and attending to unmet needs of patients before, during, and after transplantation may positively influence their experience of aHSCT.

## 1 Introduction

Multiple sclerosis (MS) is a complex autoimmune disorder affecting the central nervous system and presenting with a diverse range of neurological symptoms (1). In about 85% of persons with multiple sclerosis (pwMS) the disease initially manifests with episodic neurological symptoms, with subsequent periods of partial or full recovery, known as relapsing–remitting MS (RRMS). As the disease progresses, after 15 to 20 years approximately 60 to 70% of RRMS cases transition to the secondary-progressive course (SPMS) characterized by a gradual and persistent escalation of disability. Moreover, around 15% are diagnosed with primary-progressive MS (PPMS), where gradual worsening of neurological symptoms can be seen without initial relapses or remissions (2). Some pwMS experience a highly active form of the disease, termed ‘aggressive’ MS, with early accumulation of permanent disability. Despite early treatment with potent disease-modifying therapies (DMTs), many of these patients do not respond optimally (3–7). For these individuals, autologous hematopoietic stem cell transplantation (aHSCT) might offer a last-resort treatment option, although evidence exists indicating that aHSCT might be safe and more effective as an early treatment option instead (8–10). Currently, the European Society of Blood and Marrow Transplantation (EBMT) and the National Multiple Sclerosis Society (NMSS) recommend aHSCT for persons with RRMS and substantial breakthrough disease activity, who are younger than 45 years old, with a disease duration of up to 10 years and failure of approved DMTs (11, 12). The principle of aHSCT is the ablation of the haemato-lymphopoietic system using high-dose chemotherapy, followed by hematopoietic stem cell transplantation for immune system reconstitution. Up to 70–80% of pwMS achieve suppression of MS disease activity for up to 5 years. Recent advancements have significantly reduced treatment-related mortality rates to below 1% through improved patient selection and the use of non-myeloablative conditioning regimens (13–17). However, despite recommendations, pwMS in Germany continue to face challenges accessing aHSCT due to issues like lack of insurance coverage, insufficient knowledge about the option from treating neurologists, and restrictive indications. This creates unique difficulties for pwMS in the decision making process for aHSCT and may influence their perception of the treatment and expression of unmet needs. While previous studies explored experiences with aHSCT for other autoimmune diseases (18, 19), there is a gap in qualitative research regarding the experiences of pwMS. Up to date only one small qualitative study explored the experiences of pwMS with aHSCT in Sweden (20). To address this, we collected data from pwMS who underwent aHSCT in various European and non-European clinics, exploring individual experiences related to the decision-making process, implementation, and aftercare of aHSCT. Our study particularly focuses on understanding the impact of the treatment on pwMS’ daily life, their psycho-social health, and any potential unmet needs in supportive care surrounding aHSCT.

## 2 Materials and methods

### 2.1 Ethics statement

Ethical approval for this study (PV5770) was granted by the Ethics Committee of the Hamburg Chamber of Physicians.

### 2.2 Design

The data for this qualitative study was obtained through semi-structured interviews, which were conducted online between May 2022 and September 2022. These interviews were part of the ongoing “Patient Experiences with Multiple Sclerosis” (PExMS) project, which aims to assess the value of patient narratives in decision-making regarding the treatment of MS, in addition to evidence-based information. By providing a multimedia website based on the experiences of pwMS with diagnosis, their day-to-day life, different treatment approaches like DMTs, alternative medicine, rehabilitation, and lifestyle changes, PExMS offers a decision aid for pwMS (21). The present study yields video and audio recordings of patients’ experiences with aHSCT for MS. These resources will be used to update the website with the latest treatment options available, and contribute to the advancement of patient-centered care in MS.

### 2.3 Participants

Participants were recruited according to the maximum variation sampling strategy, to represent as many heterogeneous experiences with aHSCT as possible (22). Participants at least 18 years of age were selected concerning the following criteria: (a) place of transplantation, (b) year of transplantation (c) age at transplantation, (d) MS type, and (e) degree of disability, measured by Patient Determined Disease Steps (PDDS) (1). The aim was to obtain experience reports of aHSCT at foreign clinics in addition to transplantation experiences at different German clinics. We aimed to recruit at least one participant who underwent aHSCT more than 10 years ago and one participant with age at transplantation over 50 years. All MS types eligible for transplantation were to be included. PwMS with low and high degree of disability prior to aHSCT were recruited (PDDS <1, PDDS >6). Recruitment was done via gatekeeper selection of patients cared for at the University Medical Center Hamburg-Eppendorf (UKE), calls in social media, and asking pwMS who had received aHSCT to pass on flyers to other patients they knew who had undergone aHSCT. Persons with severe cognitive impairment or insufficient knowledge of German were excluded. After 10 interviews, we checked for variance and finally included two more participants (Table 1).

**Table 1:** Demographics and MS-related characteristics of participants (*n* = 12).

Characteristic	N (%)
Female	6 (50,0)
Age (median, range)	39,5 (32–57)
Number of participants with university degree	10 (83,3)
MS Type	
RRMS	6 (50)
PPMS	2 (16,7)
SPMS	4 (33,3)
Number of DMTs prior aHSCT (median, range)	3 (0–7)
Transplant year (median, range)	2021 (2012–2022)
Years between diagnosis and aHSCT (median, range)	8,9 (1–15)
Years between aHSCT and interview (median, range)	1 (0–10)
Age at transplantation (median, range)	36,5 (22–57)
PDDS prior aHSCT (median, range)	4 (0–7)
PDDS at time of interview (median, range)	4 (0–7)
Financing of aHSCT	
Private means (including friends and family)	5 (41,7)
Crowdfunding	3 (25)
Health insurance company	4 (33,3)

### 2.4 Data collection

A previously developed problem-centered interview guide (23) was adapted after feedback of *n* = 2 pwMS, who underwent aHSCT in Germany and Russia (24) (Additional File 1). Open-end and closed questions were used to obtain information about the experiences with MS diagnosis, daily life, and different disease management approaches focusing on the decision-making process, implementation, and recovery after the treatment. The adapted interview guide was pretested with one participant to check for appropriate length and clearness of questions. However, as no further adjustments were necessary, the first interview was included in data analysis. Prior to the interview written informed consent was obtained and information regarding demographic, MS-related, and aHSCT-related data was gathered. Interviews were conducted and recorded using video communication software, which allowed participants to take part in the interview from their homes, using infection protection measures after aHSCT, or because of mobility impairment. The interviews were conducted by a male medical student, not affected by MS, with no prior relationship to the interview partners, and who was previously trained by a research associate regarding qualitative interview techniques.

### 2.5 Data analysis

After transcription, all interviews were analyzed using the six phases of thematic analysis by Braun and Clarke (25) with the help of the program MAXQDA2022. Both deductive and inductive analytic approaches were used. After familiarizing with the data, initial codes were generated and grouped into major themes and sub-themes, followed by a constant reflection and refinement process by both coders (AS and TV) and consultation with research peers to yield clear definition and naming of the final themes. AGW, an English/German bilingual native English speaker helped to translate the quotations used in this article. We followed the consolidated criteria for reporting qualitative research (COREQ), to provide comprehensive reporting for this study (26) (Additional File 2).

## 3 Results

We interviewed 12 pwMS in this study. The length of the interviews varied between 43 and 150 min (median = 65.5). Female and male pwMS were equally represented. All MS types eligible for aHSCT were included. Two of our participants showed no signs of disability prior to aHSCT (PDDS = 0), whereas *n* = 3 showed high degree of disability (PDDS = 7). Likewise, DMT usage varied greatly, from never treated to seven different DMTs prior to aHSCT. No participant received a DMT post-aHSCT. While five participants underwent aHSCT at different clinics in Germany, three participants received aHSCT in Russia, two in Mexico, one in Italy, and one in England. Median age at transplantation was 36.5 years with one participant receiving aHSCT at the early age of 22 and one at relatively older age for aHSCT of 56. To gain insight into the experiences of pwMS who were transplanted with more rigorous conditioning regimens, we included two participants with aHSCT in 2012 and 2014. A detailed description of demographic and MS-related characteristics of all pwMS can be found in Additional File 3.

**Figure 1:**
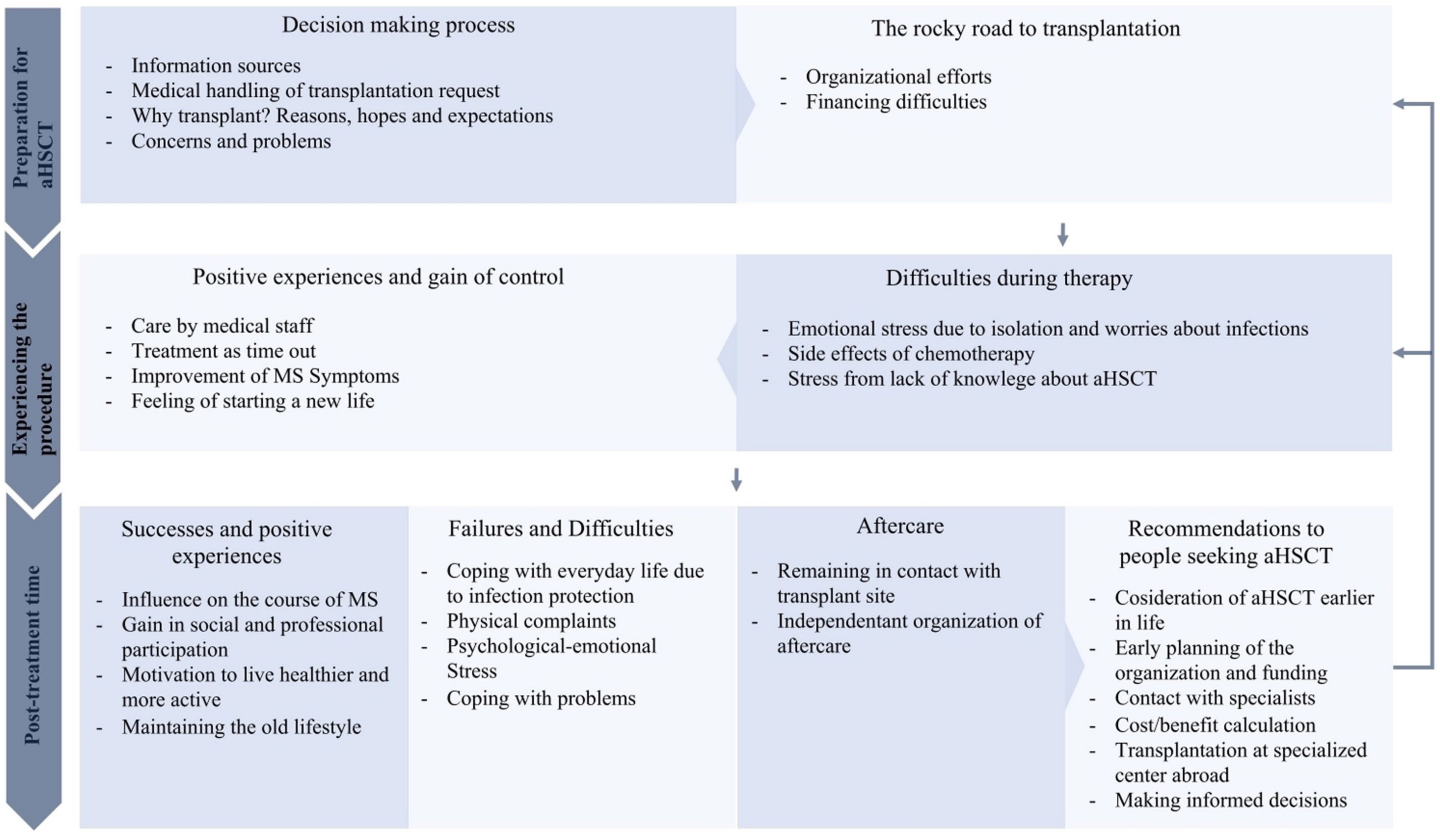
Thematic map of themes and sub-themes of pwMS’ experiences with aHSCT.

Three major themes along with their corresponding sub-themes were identified by thematic analysis: preparation for aHSCT, experiencing the procedure, and post-treatment time, as shown in Figure 1. Exemplary quotes with their corresponding themes can be found in Additional File 4.

### 3.1 Preparation for aHSCT

Preparation for aHSCT is characterized by a difficult decision-making and preparatory process. Many interviewees report many-faceted challenges ahead of transplantation, with high organizational costs and funding difficulties.

#### 3.1.1 Decision making process

The decision to undergo aHSCT was influenced by several interacting factors. Reasons for the decision varied widely among pwMS. Most interviewees stated that approved treatments had failed, leaving them with no further therapeutic options: *“My back was against the wall. The available medications did not work.”* (pwMS 12). They feared a further progression of the disease with massive restriction in everyday life: “So*, I was already close to being in a wheelchair and had the feeling that this [the DMTs] probably will not help me until I am in the wheelchair”* (pwMS 7). Often this lack of alternatives was accompanied by self-reproach for not having exhausted all options in their fight against the disease: *“[I] had actually seen this as my last hope (...) that I could hold on to and said: (...) I still have a few years ahead of me in this beautiful world. And that should not be the end. And I do not want to reproach myself 20 years from now for not having tried everything.”* (pwMS 10). Most pwMS hoped that aHSCT would stop the disease: *“My big hope, of course, was that I would no longer have any foci of inflammation in my head and that my physical fitness would remain how it was so that I could continue to do sport (…) Exercise was always the most important thing for me”* (pwMS 5). Despite a mostly realistic estimate about a possible benefit of aHSCT, some pwMS, “secretly” hoped for an improvement in their symptomatology: *“So, the hope was of course that all my disabilities would also go away - completely. And that even if they did not go away completely, that they would at least be stopped, and that I could then get back to how I was through training. But that’s not really how it works. That’s … I knew that, too. So, in my head, I already knew that it’s like that, that it only stops the disease where it is, and then maybe you can improve it a little bit through training. But sure, you never lose hope.”* (pwMS 9).

Particularly pwMS at early disease stages, with no physical impairments yet, placed great hope in aHSCT and promised themselves nearly a cure for MS. One pwMS who had not received any DMT before said, *“I knew with the transplant there was a possibility that I would get rid of the disease. And it could be for a long time or forever. And I knew I had to do it as soon as possible because so far, I have not had any permanent damage. And because of that, there was no discussion for me.”* (pwMS 3). For some interview participants, aHSCT’s reduced risk profile in recent years was particularly convincing: *“Well, the figures are relatively convincing, if you look into it a bit, it speaks very much in favor of it, (...) the mortality rate is relatively low now. It is less than 1%. Especially when you are as young as I am, nothing should really happen. And accordingly, I actually decided to do it with a clear conscience.”* (pwMS 1). In addition to scientific evidence, the stories of other pwMS and their description of positive experiences with aHSCT were also of great importance in the decision for transplantation: *“The video recordings that I had seen [of other patients](…) all said, the trade-off, what you go through and what I get back in the end in terms of quality of life. This was the basis. I would like to have that too.”* (pwMS 4). Interestingly, one interviewee reported that for her the comprehensible mechanism of transplantation was of great importance and that she deliberately preferred a myeloablative conditioning regimen because she hoped that the radicality would also be accompanied by better chances of success of the therapy: *“What convinced me is (...) that the immune system is really completely wiped out initially, and that’s why I was also glad that I was treated according to the BEAM regimen, because for me personally that somehow gave me the feeling, yes, now everything causing this inflammation is gone. You get a completely new immune system. And the thought behind it was logically comprehensible for me.”* (pwMS 6). Two pwMS indicated that the need to be able to care for their children played a role in the decision to have aHSCT: *“I was really afraid that I also - I had a child now; she was a baby at that time, too - and that I could not take care of her anymore [because of the MS].”* (pwMS 9). The reasons for deciding to transplant abroad, for many interviewees, were often rejections from German clinics, which was perceived as very frustrating: *“I thought: Well, that’s actually the same as with cancer patients and that’s actually done in every garden-*var*iety clinic. But it wasn’t like that (...) I did not fit some of the criteria [for indication].”* (pwMS 10). Others opted for transplantation abroad also because of the considered higher expertise in aHSCT in MS: *“Moscow has carried out approximately 1,500 stem cell transplants for MS so far.”* (pwMS 8).

The study participants used different sources of information in their decision-making process. In particular patient narratives seemed to have been very important. Groups on social media, video and television reports, books, and direct exchange with affected persons were used to yield information about aHSCT: *“[I] watched YouTube videos. There is one who (...) made a video diary (...). He spoke every day of what was being done. How it is done. How he is doing. And so on. I watched that.”* (pwMS 1). Information, such as studies or guidelines, was also utilized. However, it was often difficult for some study participants, especially those transplanted a longer time ago, to find or interpret information material and studies: *“And then I just looked to see if you could find studies or other reliable data on it, and there was so little high-quality data that you can find on it, but what I found did sound very good, although of course often* var*ious transplantation protocols were used, some articles were specifically about BEAM.”* (pwMS 8). Additionally, primary care physicians, neurologists, and transplant experts were consulted. Most of the interview participants who discussed their wish for transplantation with their family doctor or established neurologist felt encouraged by them, but in four cases they reported being left alone in their decision making process.. One pwMS reported: *“My neurologist here in Germany, she was completely against it at first. And only afterwards, when I had informed myself a lot and had shown her many studies and also told her that I absolutely wanted to do it, then she supported me.”* (pwMS 9). The need for information on aHSCT from their neurologists could sometimes not be met adequately or was even not met at all. *“That is unfortunately a sad experience that I have had. If I had not taken it all on by myself, nothing would have happened. So, if I had been with my first neurologist, I would be in a wheelchair by now.”* (pwMS 8). One pwMS therefore did not want to discuss her transplantation with their treating neurologist: *“I told my neurologist: I will go there [for aHSCT]. So, I did not discuss anything with him or anything. (...) I [decided] all by myself in the privacy of my own home.”* (pwMS 10).

The interviewees experienced a wide range of concerns and problems in their decision to undergo transplantation. Most of the pwMS were worried about severe side effects, complications, and death, especially in a foreign country or far away hospital, separated from their loved ones: *“But the main concern was the phase where the immune system is almost non-existent, you might catch some germ and then have massive problems in Moscow. Thousands of kilometers away from relatives and from German hospitals.”* (pwMS 3). Some pwMS worried about being separated from their children. Worries about loss of fertility were an issue for three pwMS. However, most interviewees had already completed their family planning or decided against having children and chose not to cryopreserve ovarian cells. For some, the physical limitation due to MS was also a reason to refrain from ovarian cell freezing: *“I did not want to have eggs frozen (…), when you are sitting there in a wheelchair, and you cannot move your arms and legs properly and how are you supposed to raise a child?”* (pwMS 6). Only two male pwMS opted for cryopreservation of their sperm: *“I had my sperm frozen in advance, even though I do not have a plan to have children, at least for now, it was still important to me.”* (pwMS 7). For two interviewees, concerns about treatment failure were high: *“I know there’s a certain number of people (…), where the stem cell transplant does not work at all. And I did not want to be one of those.”* (pwMS 10). Others reported a lack of confidence in medical care standards abroad: *“Mexico and Russia are not countries where you say oh, one goes there for medical treatment.”* (pwMS 2). For others especially those with fast disease progression, the biggest concern was that the transplant would not be performed quickly enough. For some interviewees, not having been able to find a contact person, who also underwent aHSCT, to share personal experiences with transplantation was a problem: *“For me, the most difficult thing was that I (...) did not have a contact person in Germany. No patient who could really tell me what the whole thing was like and how it felt. There was simply no contact person.”* (pwMS 3).

The interview participants dealt with these concerns in different ways. Some reported that positive testimonials from former patients or the perceived high standards of care in a private clinic abroad gave them relief about their worries about transplantation in foreign countries: *“It simply convinced me a bit or put me in a positive mindset when I was informed that this is actually a private clinic. They only do it for rich people “*(pwMS 10). Others coped with their worries with psychological care: *“I prepared myself very well. I looked for a psychotherapist”* (pwMS 6). For this pwMS, also spirituality was particularly important: *“I also somehow believe in something, something divine somewhere. I thought, something will somehow guide me through this.”* (pwMS 6). One pwMS, on the other hand, coped with her worries in a more destructive manner: *“First I threw myself into work and everything else. And then came the phase before the transplant, where I was rather sad and hopeless.”* (pwMS 5).

#### 3.1.2 The rocky road to transplantation

Accessing aHSCT was often described as long and stressful and is characterized by organizational effort and financing difficulties. Some interviewees reported a mostly exhausting independent search for specialized clinics: *“The work put in there, (...) from two years in total, I’d say it’s enormous. Because you always have to be on the ball. You always have to look and look and look and, above all, a lot of paperwork and then also file everything systematically.”* (pwMS 4). Often a transplantation was desired in Germany but could not be performed at the desired location mainly due to restrictive indications. Especially those with higher degree of disability had a difficult time finding a suitable transplant site, due to strict indications. Some pwMS experienced repeated rejections from clinics: *“At the same time, however, I still tried to get a transplant place in Germany. I asked in Hamburg, but was out of the question. I went to Heidelberg, (...) and there I was also rejected”* (pwMS 8). Therefore, some interviewees decided to have their transplantation carried out at a department of a foreign hospital, specialized in aHSCT in MS, which was perceived as easier with fewer restrictions and broader indications: *“Well, I registered for the stem cell transplantation in Moscow in the normal way”* (pwMS 7), *“That was relatively uncomplicated. There is this contact person in Moscow”* (pwMS 3). However, some interviewees also experienced difficulties with a transplant abroad, especially delays were reported due to travel restrictions caused by the COVID-19 pandemic: *“I had an appointment for Moscow in 2020, at the end of 2020, but then COVID came and then you could not go there anymore.”* (pwMS 8). In addition, the political situation in Russia, in view of the war in Ukraine, caused problems for one pwMS, which is why she decided not to have a transplant in Russia after having previously agreed: *“I had the confirmed treatment slot in Moscow and, as I said, I was a bit afraid for political and pandemic reasons whether I would still be able to get there.”* (pwMS 11). This circumstance led to stress and uncertainty about when and where the transplant could take place for some pwMS. For one interviewee, more than a year passed between stem cell collection and stem cell application in London, which was accompanied by considerable psychological stress: *“And that was more the problem, or the uncertainty, because you did not know when Corona would continue, and you actually want to do this transplant.”* (pwMS 12). One interviewee additionally reported that planning the stay abroad represented a burden for her*: “Then (...), I had a good month to prepare for Moscow with the visa (…) and that was tight. You really need a month.”* (pwMS 8).

Financing the transplantation was perceived as an additional burden for many interviewees. Often the financing came from their own savings, friends, or family. Three pwMS financed their aHSCT via a crowd-funding campaign: *“I had my own savings and then also started the crowdfunding campaign myself very quickly.”* (pwMS 7). In some cases, the costs could be covered in whole or in part by their health insurance companies. PwMS who had transplants abroad had to pay in advance and discuss with their health insurer about the coverage of costs. In the case of transplantations that did not take place in European countries, this posed a major challenge: *“You also have to pay for it yourself. By the way, it cost 54,500 USD (...) I had talked to my statutory health insurance beforehand and they are (...) the first and so far also the only ones who have financed a treatment outside Europe at least to a large extent [as far as I know].”* (pwMS 10).

### 3.2 Experiencing the procedure

The performance of aHSCT was perceived by many participants as a physically debilitating and psychologically stressful experience. At the same time, however, it was also regarded as a life-changing event that provided them with a sense of control over their own future.

#### 3.2.1 Difficulties during therapy

All interview participants reported substantial physical stress with the typical side effects of high-dose chemotherapy: *“Well, especially the first dose of cyclophosphamide I found very exhausting and stressful”* (pwMS 2). The most common side effects experienced were nausea, vomiting, weakness and fatigue, infections, fever, diarrhea, and hair loss. Particularly limiting for some interviewees was the perceived emotional burden of medical isolation and the constant endurance of worry about infections with accompanying complications or prolongations of the hospitalization phase: *“I was not allowed to have visitors (...). I was relatively lucky, I would say, that I only developed an infection once, so that I had to have an extra antibiotic treatment and my stay was extended by three days. But the fear is always there. That someone comes in who has a cold”* (pwMS 5). The separation from their children and overall social isolation was perceived as a mental burden: *“I was in complete isolation for a month (...) So I did not see my daughter for a month.”* (pwMS 9). Various factors aggravated the burden of isolation, for example, being abroad and the simultaneous travel restrictions due to the pandemic, but also sensory deprivations due to the isolation requirements during the high-dose chemotherapy: *“I was actually so alone there, cut off. (...) That was tough. When you see people outside walking in the sun or something. You just sat in the room and could not smell anything either.”* (pwMS 5). One participant reported mental stress, due to insufficient information provision in the informed consent process about the concept of transplantation and associated uncertainty: *“That is the stress that I have made myself there. Because that’s just, I do not know, was just my mistake to then deal with such a thing [the transplantation] without background knowledge.”* (pwMS 6).

#### 3.2.2 Positive experiences and gain of control

Interview participants dealt with the experienced loss of control in a variety of ways. Reported positive experiences during therapy, oftentimes lead to gains in control. Of particular importance was the care provided by medical staff, especially the nurses. A great deal of commitment and a willingness to help and understanding by the providing caregivers led to a reduction in mental stress for most pwMS: *“And the staff were incredibly nice. Because they had a lot of time for each patient individually. They sat down more often, when they noticed that [the patients] were a bit lonely today”* (pwMS 11). For one interviewee it was of great importance to experience psychological care during the transplantation: *“I had a psychologist at my side (...) with whom I then had contact by telephone. (...) And that already helped me.”* (pwMS 6). For many pwMS, the high degree of professionalism of the medical staff gave confidence in the medical expertise and provided security: *“So, you knew you were in good hands. And above all, I put trust in that the clinic in Moscow has already transplanted well over 2,000 patients. And that I just noticed that it was running with a routine with 20, 30 patients at a time.”* (pwMS 3). 10 of the 12 interviewees reported that contact with fellow patients, as well as friends and family, had been instrumental in helping them cope with the physically and mentally demanding situation. Many participants organized among themselves and informed themselves about the health status of fellow patients via social media. This gave the interviewees a sense of cohesion and led to stress reduction. Patients who had been transplanted abroad welcomed the intercultural exchange: *“So the whole world was there actually. And I had a fellow patient from Norway, a patient from Australia, a patient from Slovenia, a patient from Switzerland, and then we made a little messenger group and exchanged ideas.”* (pwMS 8). One participant reported that joint activities with other patients made dealing with the accompanying physical changes easier: *“We had a kind of hair-off party in Mexico. We all sat down outside, because the weather was great, and then we just shaved off all our hair.”* (pwMS 10). This gave some interviewees the feeling that they could also use the transplant as time out from their everyday lives and have time for themselves and make the best out of an otherwise stressful experience. Some pwMS said they had discovered new hobbies: *“I had a lot of time to read and make photo albums, which I had not done before.”* (pwMS 9). Others found the time abroad relaxing: *“Actually, it was more like a vacation with chemotherapy.”* (pwMS 10). Occasionally, some interview participants reported improvements in their MS symptomatology during the hospitalization period. Three interviewees reported disappearance of fatigue or decrease of paranesthesia already during transplantation: “*The most positive experience actually is, that with the therapy this fatigue is gone.”* (pwMS 3), *“What motivated me was that I noticed that my paraesthesia got a little better already during the transplant and my tingly hand got a little better.”* (pwMS 5). Some participants reported that they perceived the transplant as a life-changing event that they hoped would have a major impact on their future. They reported that they would like to start a “new life” with the aHSCT: *“[The] transplantation was like a second birthday for me, the 6^th^ of May. (...), where then the three big injections were given through the CVC. For me it was like this, you simply enjoy it now (...). I have to say it’s going up and up and up, I feel better every day.”* (pwMS 4). Other interview participants put their current euphoria into perspective and took a more pessimistic view of their future: *“Of course, one relies more (...) on the hopes one has for this therapy than on being really euphoric at the moment and saying: Everything is much better now. So, I cannot confirm that. (...) But if you are like me, ten, eleven years after diagnosis, then of course it takes time for that to settle in again.”* (pwMS 2).

### 3.3 Post-treatment time

For many interview participants, the aHSCT continues to have an impact on life after transplantation for a long time. The post-transplant period is characterized by successes and a positive impact of aHSCT on the lives of pwMS, as well as failures and difficulties. A particular issue for many interviewees seems to have been the medical aftercare, which had to be organized mostly by themselves. Retrospective revaluation of the transplantation and advice, that the pwMS would like to give to their former selves or to other pwMS emerged from these experiences.

#### 3.3.1 Successes and positive experiences

The interview partners attributed the increase in quality of life to various personal successes. For many, the positive influence of the transplantation on their MS course and their MS symptoms was a key factor. They reported about the relief due to the absence of relapses and the associated possibility of regaining physical abilities without fear of a new relapse: *“I could build myself up again without the next relapse coming immediately after the previous. I went to rehab and was able to really build up my strength. That was great.”* (pwMS 6). For pwMS, transplanted some time ago, and for those who had hardly any physical impairments before the transplantation, the thoughts no longer revolved around the MS and thus, the disease receded into the background of their lives: *“So before the transplantation, the disease actually shaped my everyday life from the morning until the evening. (...) Now, with the knowledge that I am aware that I no longer have this disease, (...) the whole thing no longer burdens me in any way and I no longer think about this disease.”* Even in some pwMS with progressive course, an at least initial stop of the progression could be achieved, which led to relief: *“I just look forward to every new day, because I think: Hey! Nothing will happen [no new symptoms]! And I feel like everyone my own age, I say, who is my age. Nothing’s getting worse again. And that’s just a beautiful thing.”* (pwMS 10). Many interviewees additionally reported improvements in their MS symptoms, which they attributed to the transplant. They often described a decrease in sensitivity problems, an improvement in gait or walking distance: *“I drink cappuccino and before the treatment, I was no longer able to transport a filled cappuccino cup from the kitchen to the living room, it spilled over and now I can do it again.”* (pwMS 8). This removes burdens and makes it easier to cope with everyday life. Some participants also reported cognitive improvements such as an increase in concentration or disappearance of fatigue. Sometimes minor success made a big difference for some interviewees: *“But the one improvement that I have, where I can objectively say that it is certainly due to the stem cell transplant, is my (regained) heat tolerance (now being able to enjoy the sun).”* (pwMS 7).

Several aspects favored the gain in social and occupational participation, which most interview participants experienced after the transplantation. In addition to the direct influence of the aHSCT on the disease described above, the discontinuation of MS medication, omission of recurring doctor’s appointments, social pressure due to frequent sick leaves, and the renunciation of mobility aids also led to increased social participation. *“I enjoy it all much more than before. (…) I had to take care of medication and doctors. (…) you always have to justify yourself. Why are you on sick leave again and not there again?”* (pwMS 5). Particularly valued was the possibility of caring for their own children, which the transplant has facilitated: *“I got a life back. And that is worth quite a lot. I can be there for my child. That’s the best thing ever.”* (pwMS 6). One interviewee reported that she was able to get pregnant after the transplant: *“I also had two healthy children after the transplant, which I’m totally grateful for, because that was just one of my biggest fears, that it would not be possible.”* (pwMS 5).

Many interviewees reported that the transplant motivated them to live healthier and more active lives. Often this was evidenced by a change in health awareness and outlook on life. Whereas one participant reported wanting to prepare as best as possible for aHSCT: *“I need to go into this stem cell transplant physically fit, so I do not have any sequelae in the transplant itself.”* (pwMS 3), others said that they experienced a change in health consciousness because of the transplant: *“And then, of course, you have a completely different attitude to life, (...) You just know it’s not going to get worse, it can only get better.”* (pwMS 2). Especially experiencing and enduring physical exertion through aHSCT, but also the financial aspect, motivated many participants to behave more consciously and healthier: *“I cannot go through such an expensive transplant and then I do not take care of myself; then I really have to give 100%.”* (pwMS 7). This manifested itself, for example, in increased physical activity: *“After the transplant, I then said, no, not just lots of sport, but really structured: (...) 40 min of strength training and really a different muscle group every day.”* (pwMS 7), some participants adjusted their eating habits: *“I now actually never eat any convenience products, but concentrate totally on wholefood nutrition (...) I also cut out sugar completely.”* (pwMS 7). In addition, the avoidance of noxious substances played a role: *“I abruptly stopped smoking”* (pwMS 3). For some interviewees, aHSCT led to deceleration of life, resulting in stress reduction: *“I also filter more. What is important now? What do I actually need to do now? And what is stress that I’m adding to myself that’s making me sick?”* (pwMS 5). For some interviewees, aHSCT enabled them to continue with their old habits: *“Otherwise, my lifestyle itself did not change much (…) so I will just continue the healthy lifestyle.”* (pwMS 8).

#### 3.3.2 Failures and difficulties

Study participants experienced a variety of health failures and difficulties after transplantation. Mostly, these were physical complaints in close temporal connection to the aHSCT. Our Interviewees mainly described side effects of chemotherapy and its indirect effects such as infections, sometimes aggravated by already increased infection risk due to disabilities: *“I had gotten a bladder infection [back] in Germany. That’s when the catheter was taken out”* (pwMS 10). Three interview participants reported that they felt that new MS symptoms had developed despite aHSCT. For two of these participants, shortly after aHSCT, it was difficult to distinguish whether the symptoms already existed before and have become worse due to the transplantation, or whether they were new symptoms:” *It is constant, the fighting back, (...) I actually walk worse since then, (…) of course you need to rest and this rest does not exactly make it easier in terms of mobility.”* (pwMS 2). One interviewee reported that the initial stop of the progression did not last and that four years later the symptoms were worsening again: *“It is slowly getting worse. Cognitively or just with the bladder-bowel story, from walking I notice that the distance has become less again.”* (pwMS 6). Other complaints included diarrhea, hemorrhoidal disease, weight loss, skin rashes, and joint pain.

In addition to physical discomfort, interviewees also reported psychological and emotional stress shortly after transplantation. Especially the worry about infections caused some participants great concern and uncertainty: *“which is exhausting (...) to be so on guard. So especially in the beginning, what do I do if I get a fever at night?”* (pwMS 8). An additional burden for some participants was the COVID-19 pandemic. The isolation associated with infection control was often accompanied by feelings of loneliness; this was a major challenge for several interview participants: *“I hardly saw any friends for half a year. I did not go shopping; I did not go to events. No one actually came into our house without a mask and gown (...). There were also a lot of reflective days where I thought, wow, that was hell and back.”* (pwMS 5). Some interview participants also reported sleep disturbances, depression, or mental overload. For one participant, the years following transplantation were filled with constant doubt as to whether the therapy was successful: *“There you are always wavering: did it work; do I notice something new; is this a new symptom; do I have another relapse. That took several years.”* (pwMS 9).

For many interviewees, the sudden realization of their own physical weakness and fatigue after transplantation due to deconditioning was a surprise. The discharge into the home environment and the associated coping with everyday life made some of the interviewees aware of their own weakness: *“That was the beginning of a somewhat harder time, of course, because then you are extremely cautious. I did not go out at all during that time and was so weak that it was really difficult for me to get around.”* (pwMS 7).

Participants coped with these problems in different ways. They used a variety of strategies and a range of medical and social interventions. All participants reported using medical measures to protect against infection. One participant reported passive vaccination as protection against COVID-19 and not having the children cared for at daycare. Many reported following hygiene rules required when interacting with other people, pets and during housework. Other medical measures included physiotherapy and rehabilitation, which made the time after transplantation easier for many participants: *“I went straight to follow-up treatment (...). And then trained like crazy for weeks.”* (pwMS 6). Regarding social measures, the participants reported that the help of friends and family had clearly helped them: *“I was lucky that (...) people from the family were always [there and] brought me to the [medical facility], because I definitely could not drive a car myself yet.”* (pwMS 5). Especially those with higher degree of disability seemed to rely more on their social network after transplantation. Government assistance in the form of parental assistance, household help, or transportation services was also used: *“Also got household help through the health insurance. That has really been worth its weight in gold.”* (pwMS 1). In addition to resting, perseverance, and the positive reassessment of the health situation already described, the relativization of long-term risks that can accompany aHSCT were a coping strategies used by many interviewees: *“The risks are limited. I would say I also have a risk when I’m driving on the German autobahn.”* (pwMS 4).

#### 3.3.3 Aftercare

A significant topic for most interview participants represented the medical aftercare following aHSCT. In general, it can be said that there was no uniformly regulated follow-up for medical care after transplantation. For most interviewees, the transplant center remained a contact for regular check-ups. This was also the case for transplantation abroad. For the most part, these patients were in contact with the doctors at the transplant center abroad via e-mail or messenger programs: *“So a doctor’s consultation in England* via *Zoom, that’s very quick. State your credit card number and that works beautifully.”* (pwMS 12). One participant traveled abroad annually for checkups, which involved increased time, costs and effort, *“Until Corona I went [to Florence] once a year, every year (…) after the transplant. I had an MRI done once a year and then sent it there in advance”* (pwMS 9). For the most part, medical follow-up was organized independently by the pwMS themselves Often, neurological follow-up was done by the resident neurologist. Some participants turned to a resident oncologist, with whom some interviewees were not satisfied: *“The aftercare [at the] oncologist I must say, was very adventurous. (...) I’m a relative exotic [case] in oncology and I did not really feel that I belonged there, or that I had been acknowledged.”* (pwMS 4).

#### 3.3.4 Recommendations to people seeking aHSCT

Retrospective reflection led all interviewees to wish for changes in their own aHSCT. Seven pwMS, mostly those with higher degree of disability, wished they had heard about the transplant option earlier and would have preferred to have it performed earlier to prevent permanent physical damage. Some would have liked to have been informed about the organizational effort at an early stage and would advise others not to underestimate it: *“It’s not just the transplant itself, but that the whole thing also brings up a whole bunch of problems, workwise, financially.”* (pwMS 5). The financing of the aHSCT was especially critical: *“Take care that you get the money together. (...) Anyone can be affected, whether their purse is big or not so big. And it should not depend on your wallet whether you get reasonable, good treatment or not.”* (pwMS 10). Some participants advise early contact with MS specialists: *“So especially with MS, my feeling is that it does not work the same way as with the normal cold, where you go to the doctor and he knows his medication, (...). It is not enough to go to the normal neurologist around the corner. One should really deal with the subject and also visit experts somewhere.”* (pwMS 12). Some interviewees advise not to underestimate the treatment and would recommend others to carry out a strict cost/benefit calculation: *“I am definitely of the opinion that one should always weigh up the benefits and the risks. (...) What I (...) got is that many consider [aHSCT] even with mild MS and I think there the ratio is off.”* (pwMS 6). Therefore, it was important for many interviewees to make an informed decision: *“To read quite a lot, to form your own opinion. That’s my advice for everybody.”* (pwMS 9). One pwMS seems to have been very convinced of his transplantation abroad and, in retrospect, would have it done there again: *“If you have the money, by the way, I would not take Germany, but rather Mexico or Moscow, because there is simply much, much more experience available than at the moment in 2022 in Germany.”* (pwMS 10).

## 4 Discussion

AHSCT is a promising and effective treatment option for people suffering from highly active MS with possible severe short-term side effects and risks. Little is known about how pwMS, who have undergone aHSCT, experience the decision-making process, the procedure itself, and the recovery after transplantation. A study with eight pwMS undergoing aHSCT from 2004 to 2007 at Uppsala University Hospital was recently published (20). Similar to their findings, in our study pwMS reported a feeling of losing control before and regaining it after transplantation, a perception of having the possibility to start a new life and re-establishing a state of health was described. Whereas the findings of Tolf et al. (20) mainly focused on the time quite a while after aHSCT (mostly more than 10 years ago) and its impact on daily life, our study also focuses on the lived experiences regarding the preparation, decision making process and the implementation of the procedure itself and the detection of unmet needs. This study offers insight into the experiences of 12 pwMS who have undergone aHSCT at different transplantation sites. We observed a difficult preparatory and decision-making process for most study participants. Whereas high, but realistic expectations about the treatment outcome were common, some pwMS, mostly patients with no prior DMT, expected aHSCT to be nearly a cure for MS. This is in line with previous findings, showing, that 79% of pwMS, not yet been treated with aHSCT, expect a complete stop of disability progression after aHSCT (27). We found that a dissatisfactory experience with previous DMTs and their efficacy was the main motivator to undergo aHSCT. The internet, television, social media, and patient narratives, all potentially untrustworthy sources, were mostly used as information sources, although expert opinion was preferred. PwMS generally seem to gather information on MS and aHSCT mainly online (27, 28). Given that pwMS may be more susceptible to framing effects in communication (29), and considering our observation of some pwMS expressed dissatisfaction with discussing aHSCT with their treating neurologist, it suggests a potential deficiency in the objective information provision regarding aHSCT for certain pwMS. Some pwMS felt alone in their decision and wished for more support in the preparatory process. This underscores the necessity for treating neurologists to acquire comprehensive knowledge about aHSCT procedures or to promptly refer patients to specialized clinics where they can receive appropriate treatment counseling. This applies especially also for the aftercare.

We identified several challenges and concerns pwMS had to deal with in their decision-making process. Fear of side-effects of chemotherapy, complications and death, separation from their friends and family, and fear of treatment failure experienced especially by pwMS with PPMS were commonly observed. One participant felt unprepared for aHSCT resulting in distress, showing the need for proper patient education as part of the decision-making process. We know from oncological practice, that educational programs about treatment procedures may prevent transplantation-related psycho-emotional stress (30). A standardized protocol for decision-making and informed consent process for pwMS with aggressive MS was proposed by Bertolotto et al. (31), entailing several physician encounters and discussions prior to aHSCT, with optional offering of psychological support and including family members in the process, yet this protocol seems not to be implemented at specialized transplantation sites yet.

We also found financial and organizational difficulties in the preparational process, as patients often had to seek transplantation in foreign countries, with the costs not being covered by German health insurance. Financial toxicity—the objective financial burden of medical expenses—can negatively impact clinical outcomes and quality of life (QoL) after transplantation. This underscores the need for financial support for pwMS undergoing aHSCT (32).

Regarding the impact of aHSCT on the life of pwMS, we observed both physical and psychological effects during hospitalization and even long after patients were discharged. Different conditioning regimens seemed not to have affected treatment experiences in our study, although based on the small sample size this cannot be regarded representative. Various coping mechanisms were observed in response to several stressors during hospitalization and after discharge. Alongside physical stressors such as chemotherapy side-effects, common psychological stressors included the emotional burden of isolation, lack of social contact, and uncertainty about treatment time and prognosis. Repeatedly mentioned coping strategies included, taking a positive perspective on present situations and thereby altering one’s outlook on life. Seeking social support from family members and fellow patients was also frequently employed. Similar desires for increased connection with other patients have been reported in qualitative studies on oncological patients undergoing hematopoietic stem cell therapy (33). The importance of social support and its positive effect on QoL, distress, and survivorship after transplantation is well documented for oncological patients (34–36), indicating the need to promote its implementation in clinical practice regarding aHSCT for pwMS.

Interestingly the change toward a positive outlook on patient’s life and health might be linked to a desire to take control over one’s disease management. AHSCT might be a motivator for pwMS to live a healthier life. The aspiration to actively enhance one’s health might be associated with a belief in self-efficacy, which refers to the perception of having the capability to overcome particular challenging circumstances (37). The perception of self-efficacy, which predicts motivation for health behavior change (38), is also linked to the perception of enhanced physical and psychological health status (39), consequently influencing the overall QoL (40). This might offer potential for supportive care, to use measures actively promoting self-efficacy of pwMS undergoing aHSCT.

Our interviewees reported immediate and sustained improvements in symptomology. Besides the true efficacy of aHSCT, one possible explanation for immediate relief might be the placebo effect. Measuring the placebo effect of intensive treatments is difficult and poses ethical considerations (41). Previous randomized controlled trials focused on comparing aHSCT to approved DMTs (42). Many factors positively influence the power and duration of the placebo effect, and more invasive procedures are suggested to have a larger placebo effect (43). High treatment expectations and patient knowledge, the option of choice, and self-efficacy are predictors for larger effects (44–46). Regarding the sustainability of the placebo effect over time, results from meta-analyses of placebo effects in surgical trials reveal a significant and lasting improvement in the placebo group, persisting even up to a year after a sham surgery (47, 48). In pwMS a trial with Fampridine indicates that the placebo effect is not persistent after 6 months and positively correlates with the expectations of treatment outcome (49). Considering the invasiveness of the procedure, pwMS’ high expectations of aHSCT (27), and its influence on self-efficacy, we suppose that an accompanying placebo effect of aHSCT might be significant. Determining its duration though remains uncertain. Those pwMS who financed aHSCT through personal funds might be particularly susceptible to this effect due to their substantial financial investment, which could lead to false assumptions of treatment efficacy by pwMS. Standardly assessing treatment expectations prior to aHSCT, but also during the procedure might be helpful in clinical practice to predict the need for support.

Medical aftercare routinely had to be organized by patients on their own, especially for those who received their transplant in a foreign clinic, where prolonged safety management but also rehabilitation therapy after aHSCT cannot be offered. While for most pwMS, the transplantation site remained a specialist contact partner, many pwMS referred themselves to oncologists, physiotherapists, and neurologists. Some pwMS experienced difficulties finding a suitable healthcare provider, highlighting the unmet need for support after aHSCT for pwMS. In some cases, study participants, mostly with few disabilities, did not rely on any medical aftercare other than having their bloodwork done by their general practitioner.

The EBMT suggests that it is essential to undergo a comprehensive assessment of all aspects of care at least once a year, conducted by healthcare professionals knowledgeable in MS. Therapists should be mindful of the late effects of aHSCT, as well as the possibility of MS relapse and progression (50).

### 4.1 Strengths and limitations

This qualitative study has the following strengths and limitations: first, there is a risk of recall bias as some interview partners underwent aHSCT a long time ago, which might distort their view of retrospective events. However, the median time between aHSCT and the time of the interview was 1 year. Second, our sampling method partially involved sampling participants through social media, which could lead to bias toward a more positive presentation of experiences. To counteract this risk, a gatekeeper sampling strategy was applied. PwMS who had negative experiences were carefully selected by an experienced researcher (CH). Third, our sample might not reflect a typical population of pwMS eligible for transplantation due to their higher educational and in some cases higher economic status. Furthermore, the sample might consist of more outgoing and agreeable extroverted individuals since participants were aware that videos of the interviews would be published on our website. With regards to our sampling strategy, our goal to interview a heterogenous group, that covers a maximum variation of different pwMS who underwent aHSCT, recruitment was achieved. The maximum variation sampling strategy yielded wide-ranging insights into experiences from different transplant locations and from pwMS with different MS courses and varying degrees of disability. By providing a detailed description of our research methodology and the characteristics of our study participants, the reader gains the ability to determine whether the findings are transferable to other settings (51, 52). Fourth, interviews were conducted and recorded using video communication software, this might allow our participants to feel comfortable while sharing experiences of personal stories (53). Fifth, after the frequent recurrence of themes in subsequent interviews, we believe data saturation has been reached. However, owing to the substantial richness of patient backgrounds and the complexity of motivations, determining this has proven to be challenging. Lastly, the researcher conducting the interviews presented as a male not affected by MS, which could have had an impact on the relationship with the interviewees, influencing the richness of the interview outcomes both positively and negatively.

## 5 Conclusion

This study provides insights into the experiences of pwMS undergoing aHSCT. We have demonstrated that aHSCT had a meaningful impact on people’s physical and psychological health, as well as social life, significantly influencing their QoL. We identified unmet needs for support in the preparatory process, during therapy, and in medical aftercare. Therefore, it is essential for clinical practice to offer a comprehensive range of support for pwMS undergoing aHSCT, including peer contacts, patient education, implementation of standardized decision-making and informed consent process protocols, job coaching, and rehabilitation programs. Future research is needed to determine the most effective ways to implement support for pwMS undergoing transplantation.

## Data availability statement

The raw data supporting the conclusions of this article will be made available by the authors, without undue reservation.

## Ethics statement

The studies involving humans were approved by Ethics Committee of the Hamburg Chamber of Physicians. The studies were conducted in accordance with the local legislation and institutional requirements. The participants provided their written informed consent to participate in this study.

## Author contributions

TV: Conceptualization, Data curation, Formal analysis, Investigation, Methodology, Visualization, Writing – original draft, Writing – review & editing. AS: Conceptualization, Data curation, Formal analysis, Funding acquisition, Methodology, Project administration, Supervision, Writing – original draft, Writing – review & editing. FF: Formal analysis, Writing – review & editing. JR: Investigation, Writing – review & editing. AW: Validation, Writing – review & editing. VH: Formal analysis, Writing – review & editing. CH: Conceptualization, Funding acquisition, Methodology, Project administration, Supervision, Validation, Writing – review & editing.
