# Supplementary material for: “A second birthday”? Experiences of persons with multiple sclerosis treated with autologous hematopoietic stem cell transplantation—a qualitative interview study
Source: Front Neurol. 2024 May 1;15:1384551. doi: 10.3389/fneur.2024.1384551 (PMC11094363; doi:10.3389/fneur.2024.1384551)
Supplement: Supplementary file 1 [file Data_Sheet_1.docx]

| 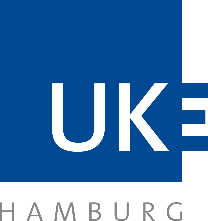 | Patient Experiences of Multiple Sclerosis (PExMS) Project.  Subproject: Interviews on stem cell transplantation |
| --- | --- |

**Additional file 1**

**Interview guide**

**"Experiences of people with MS with the disease in everyday life and with therapies".**

Pseudonym ____________________________________ Place, date_____________________

Start of interview: __________________ (h:min)

End of interview: ___________________ (h:min)

**Interview introduction**

"We developed the website [www.ms-erfahrungen.de](http://www.ms-erfahrungen.de). Here you can find videos in which MS patients speak about their personal experiences with the disease.

The aim of the project is to find out what **personal experiences people with MS** have **in everyday life.** The **focus of** the interviews is primarily on **MS therapies**. This includes **immune medication**, **stem cell transplantation**, **rehabilitation** and also **sports** and **exercise**, **nutrition** and **stress management**.

For this website, we interview MS patients and record them on video and audio. We then uploaded these videos on a website that people with MS can use as a source of information.

Since we would like to use your statements for the website, we would like to record the conversation by **video and audio.** Your data will of course be treated confidentially and anonymously. Your name will be replaced by a pseudonym. Nevertheless, **video excerpts** will be **available on the internet** where you **can be recognized**.

**Before the publication of** the videos you will receive from me the **text of** what was said from the video excerpts. Then you have the possibility to check what you said and also to be able to say that you don't want the publication of the passages sent to you after all. **After the interview,** you can also tell me if there are any parts that should not be published.

As I mentioned before, this interview will mainly be about your experiences with MS in your everyday life and MS therapies. I just ask you to be **open about your opinion and experiences,** because there is no right or wrong answer."

**🡪 Start recording!**

| **Guiding question (narrative prompt)** | **Check - Was that mentioned?**  **Memo for possible follow-up** | **Specific questions** |
| --- | --- | --- |
| I. I would like you to tell me about your life with MS. It is best to start with how you came to be diagnosed with MS. | - Symptoms and signs before diagnosis - Investigations - Diagnosis |  |
| II. please tell me if you have told those around you that you have MS. If so, what was it like when you told them? | - Who did the person confide in? - When did the person tell you about MS? | - How did you tell those around you? - When did you tell them? - Who did you tell? - With whom have you considered telling or not telling about your condition? |
| III. please tell me about your experience with medications for MS. | Only acute treatment of the relapse?   - Cortisone therapy - Plasmapheresis   Immunotherapies   - Glatiramer acetate (Copaxone, Clift®) - Inteferon - beta (Avonex™, Refib®, Plegridy™, Betaferon®, Extavia®) - Dimethyl fumarate (Tecfidera®) - Teriflunomide (Aubagio®) - Alemtuzumab (Lemtrada®) - Daclicumab (Zinbryta®) - Fingolimod (Gilenya®) - Mitoxantrone (Novantron®) - Natalizumab (Tysabri®) - Cladribine (Mavenclad®) - Ocrelizumab (Ocrevus®) - Ozanimod (Zeposia®) - Ponesimod (Ponvory®) - Ofatumumab (Kesimpta®)   Handling and side effects of the immune drugs | Question for sufferers who have taken immune medications and who have not:  Can you tell me about what that was like back then, when you had to decide whether to start a therapy or not?  Question for sufferers who have taken immune medications:   - Please tell me how you are taking the medication and how you are coping with taking it? - Have you had any problems with the medication(s)?   Question for affected individuals who have not taken immune medications:  How did you feel about not taking immune medications? |
| IV. Please tell me about your experience with stem cell transplantation (AHST). | Decision-making process   - Information sources (factual information, medical personnel, experiences of others with MS) - Neurologists' handling of the desire to perform an aHSCT. - Hopes and expectations for the aHSCT - Worries with the decision   Implementation of the AHST   - When / Where? - Protocol of the aHSCT (MIST, etc.) - Difficulties & positives during the implementation   Time after therapy   - Effect/benefit after 3 months & 12 months - Follow-up with a hematologist and neurologist - Dealing with knowledge of long-term risks, e.g., cancers, secondary immune diseases such as hypothyroidism   Advice to others with MS | Can you tell me about what it was like for you back then when you decided to **have a stem cell transplant (aHSCT)**?   - How did you find out about aHSCT? - Did you have contact with people treated with aHSCT beforehand? How important was this contact for you when making your decision? - Did you discuss the therapy with your doctors? If yes, how was that? If no, what prevented you from doing so? - What has been your experience with neurologists in this regard? - Was fertility an issue in the approach to therapy? Were you informed about this? - Did you feel that your consulting physicians were knowledgeable about the therapy? - What convinced you most to choose an aHSCT and why? - What were your hopes and expectations for AHST? - What concerns did you have when making your decision?   Please tell me how you got **access to aHSCT** and **how it was performed**?   - When was the aHSCT done and who performed it? - Do they know according to which protocol the AHST was performed? - How did you deal with difficulties - e.g. side effects or loneliness and boredom - during therapy? - What positive experiences have you had during therapy?   How did you experience the **time after therapy**?   - In the first 3 months, the first year after that? - Did you have any problems after the aHSCT? - How did you **organize** the **follow-up after your aHSCT**? (Do you have a hematologist and neurologist as contact persons to ensure a regulated aftercare?) - How do they deal with the **knowledge of possible long-term risks**?   Would you **do** the **aHSCT again**?  What **advice** would you give to others who are interested in aHSCT? |
| V. Besides MS medications, there are other therapies that can be done. These include alternative therapies or lifestyle changes.  If you have tried anything, please tell me about it. | Lifestyle Measures   - Nutrition, food supplements - Exercise, sports (physiotherapy) - Relaxation / stress management (psychotherapy)   Alternative therapies, e.g.:   - Homeopathy - Ayurveda - Acupuncture - Cranio-Sacral Therapy - Treatments on biological basis (incense) | - What have you already tried and how did you feel about it? - How did you decide to do this? - **Has aHSCT had an impact on your lifestyle?** |
| VI. What has been your experience with rehab? | - Outpatient? - Stationary? | If rehab was done:   - Can you tell me about how you decided to go to rehab? - What impact did the rehab have on you?   If no rehab was done:   - Was this topic ever on the agenda? - If so, can you tell me about how you decided against rehab? |
| VII Could you please tell me about your daily life with MS. | - Work and profession - Occupational disability and retirement - Social life - Pregnancy, birth, children - Adaptation, aids - Mobility, Travel |  |
| **Closing questions** | | |
| What in your life has been most helpful in dealing with MS? | | |
| What has made it most difficult for you to deal with MS? | | |
| What prompted you to participate in this interview? | | |
