# Supplementary material for: “A second birthday”? Experiences of persons with multiple sclerosis treated with autologous hematopoietic stem cell transplantation—a qualitative interview study
Source: Front Neurol. 2024 May 1;15:1384551. doi: 10.3389/fneur.2024.1384551 (PMC11094363; doi:10.3389/fneur.2024.1384551)
Supplement: Supplementary file 2 [file Data_Sheet_2.docx]

**Consolidated criteria for reporting qualitative studies (COREQ): 32-item checklist**

Developed from:

Tong A, Sainsbury P, Craig J. Consolidated criteria for reporting qualitative research (COREQ): a 32-item checklist for interviews and focus groups. *International Journal for Quality in Health Care*. 2007. Volume 19, Number 6: pp. 349 – 357

**DOMAIN 1: Research team and reflexivity**

| **Items** | **Guide question/description** | **Reported on Page #** |
| --- | --- | --- |
| *Personal characteristics* | | |
| Interviewer/facilitator | Which author/s conducted the interview or focus group? | P. 4:  “The Interviews were conducted by a male medical student, not affected by MS, […] who was previously trained by a research associate regarding qualitative interview techniques.” |
| Occupation | What was their occupation at the time of the study? |  |
| Gender | Was the researcher male or female? |  |
| Experience and training | What experience or training did the researcher have? |  |
| *Relationship with participants* | | |
| Relationship established | Was a relationship established prior to study commencement? | P. 3:  “[…] with no prior relationship to the interview partners” |

**DOMAIN 2: Study design**

| **Items** | **Guide question/description** | **Reported on Page #** |
| --- | --- | --- |
| *Theoretical framework* | What methodological orientation was stated to underpin the study? e.g. grounded theory, discourse analysis, ethnography, phenomenology, content analysis | P. 4:  “All interviews were analyzed using the six phases of thematic analysis by Braun and Clarke [..] Both deductive and inductive analytic approaches were used.” |
| *Participant selection* | | |
| Sampling | How were participants selected? | P. 3:  “Participants were recruited according to the maximum variation sampling strategy, (...) Participants at least 18 years of age were selected concerning the following criteria: a) place of transplantation, b) year of transplantation c) age at transplantation, d) MS type, and e) degree of disability, measured by Patient Determined Disease Steps (PDDS) [1]. The aim was to obtain experience reports of aHSCT at foreign clinics in addition to transplantation experiences at different German clinics. We aimed to recruit at least one participant with aHSCT more than 10 years ago and one participant with age at transplantation over 50. All MS types, eligible for transplantation, were to be included. PwMS with low and high degree of disability prior to aHSCT were recruited (PDDS < 1, PDDS > 6). Recruitment was done via gatekeeper selection of patients cared for at the University Medical Center Hamburg-Eppendorf (UKE), calls in social media, and asking pwMS who had received aHSCT to pass on flyers to other patients they knew who had undergone aHSCT. Persons with severe cognitive impairment or insufficient knowledge of German were excluded. After 10 interviews, we checked for variance and finally included two more participants.” |
| Sample size | How many participants were in the study? | P. 4:  “We interviewed 12 pwMS in this study.” |
| *Setting* | | |
| Setting of data collection | Where was the data collected? e.g. home, clinic, workplace | P. 3:  “Interviews were conducted and recorded using video communication software, which allowed participants to take part in the interview from their homes, while still in isolation after aHSCT or because of mobility impairment.“ |
| Description of sample | What are the important characteristics of the sample? e.g. demographic data, date | P. 19:  Table 1. Demographic and MS-related characteristics of participants. |
| *Data collection* | | |
| Interview guide | Were questions, prompts, guides provided by the authors? Was it pilot tested? | P. 3:  “A previously developed problem-centered interview guide (22) was adapted after feedback of n=2 pwMS, who underwent aHSCT in Germany and Russia (23) (additional file 1). Open-end and closed questions were used to obtain information about the experiences with MS diagnosis, daily life, and different disease management approaches focusing on the decision-making process, implementation, and recovery after the treatment. The adapted interview guide was pretested with one participant to check for appropriate length and clearness of questions. However, as no further adjustments were necessary, the first interview was included in data analysis.“ |
| Audio/visual recording | Did the research use audio or visual recording to collect the data? | P. 3:  “Interviews were conducted and recorded using video communication software“ |
| Duration | What was the duration of the inter views or focus group? | P. 4:  “The length of the interviews varied between 43 and 150 minutes (median = 65.5).” |
| Data saturation | Was data saturation discussed? | P. 14:  “Fifth, after the frequent recurrence of themes in subsequent interviews, we believe data saturation has been reached. However, owing to the substantial richness of patient backgrounds and the complexity of motivations, determining this has proven to be challenging.” |

**DOMAIN 3: Analysis and findings**

| **Items** | **Guide question/description** | **Reported on Page #** |
| --- | --- | --- |
| *Data analysis* | | |
| Number of data coders | How many data coders coded the data? | P. 15:  “TV collected the data, with the help of JR, and conducted the thematic analysis with AS and support from FF and VH in the analysis discussion.” |
| Derivation of themes | Were themes identified in advance or derived from the data? | P. 4:  “Both deductive and inductive analytic approaches were used.“ |
| Software | What software, if applicable, was used to manage the data? | P. 4:  “(…) with the help of the program MAXQDA2022” |
| *Reporting* | | |
| Quotations presented | Were participant quotations presented to illustrate the themes/findings? Was each quotation identified? e.g. participant number | In the section “Results” and in additional file 4 we have presented quotations for each theme and identified each quotation with a participant number. |
| Clarity of major themes | Were major themes clearly presented in the findings? | Figure 1 illustrates major theme and corresponding sub-themes (minor themes). Furthermore, each theme was described in detail in the results part of this study. |
| Clarity of minor themes | Were minor themes clearly presented in the findings? |  |
