# Supplementary material for: “A second birthday”? Experiences of persons with multiple sclerosis treated with autologous hematopoietic stem cell transplantation—a qualitative interview study
Source: Front Neurol. 2024 May 1;15:1384551. doi: 10.3389/fneur.2024.1384551 (PMC11094363; doi:10.3389/fneur.2024.1384551)
Supplement: Supplementary file 3 [file Data_Sheet_3.docx]

| Pseudonym | Age | Gender | Highest education | Year of diagnosis | MS-type | DMTs | Place of aHSCT | Year of aHSCT | Funding | Conditioning | Disease activity after aHSCT |
| --- | --- | --- | --- | --- | --- | --- | --- | --- | --- | --- | --- |
| AHST-01 | 32 | female | Universtity | 2007 | RRMS | 3 | Hamburg | 2022 | Insurance | Cyclophosphamide | stable, no relapses, no new MRI lesions |
| AHST-02 | 44 | male | Universtity | 2010 | SPMS | 3 | Puebla | 2021 | Private | Cyclophosphamide | possibly progressing (decreased walking distance, spasticity)* |
| AHST-03 | 40 | male | Universtity | 2018 | RRMS | 0 | Moscow | 2019 | Private | Cyclophosphamide | stable* |
| AHST-04 | 48 | male | Universtity | 2017 | PPMS | 1 | Hamburg | 2022 | Insurance | Cyclophosphamide | progressing (decreased walking distance), no relapses, no new MRI lesions |
| AHST-05 | 32 | female | vocational education | 2007 | RRMS | 2 | Hamburg | 2012 | Insurance | BEAM + ATG | stable, no relapses, no new MRI lesions, no progression |
| AHST-06 | 37 | female | vocational education | 2008 | RRMS | 7 | Münster | 2018 | Private | BEAM + ATG | possibly progressing (decreased walking distance, increasing incontinence)* |
| AHST-07 | 39 | male | Universtity | 2007 | SPMS | 3 | Moscow | 2021 | Crowdfunding | Cyclophosphamide | stable* |
| AHST-08 | 57 | female | Universtity | 2017 | PPMS | 2 | Moscow | 2021 | Private | Cyclophosphamide | stable* |
| AHST-09 | 43 | female | Universtity | 1999 | RRMS | 3 | Florence | 2014 | Private | BEAM + ATG | stable* |
| AHST-10 | 43 | male | Universtity | 2007 | SPMS | 2 | Puebla | 2020 | Insurance | Cyclophosphamide | stable* |
| AHST-11 | 32 | female | Universtity | 2014 | RRMS | 3 | Heidelberg | 2021 | Crowdfunding | Cyclophosphamide + ATG | stable* |
| AHST-12 | 37 | male | Universtity | 2014 | SPMS | 3 | London | 2021 | Crowdfunding | Cyclophosphamide + ATG | progressing, no relapses, no new MRI lesions |
| aHSCT = autologous hematopoietic stem cell transplantation, DMT = disease-modifying therapy, RRMS = relapsing remitting multiple sclerosis, SPMS = secondary progressive multiple sclerosis, PPMS = primary progressive multiple sclerosis, BEAM = BCNU Etoposide Cytarabine Melphalan  * self-reported data | | | | | | | | | | | |

Additional file 3: Demographic and MS-related characteristics of individual pwMS
