# Supplementary material for: “A second birthday”? Experiences of persons with multiple sclerosis treated with autologous hematopoietic stem cell transplantation—a qualitative interview study
Source: Front Neurol. 2024 May 1;15:1384551. doi: 10.3389/fneur.2024.1384551 (PMC11094363; doi:10.3389/fneur.2024.1384551)
Supplement: Supplementary file 4 [file Data_Sheet_4.docx]

Additional file 4: themes and sub-themes with exemplary quotes

| themes/ sub-themes | exemplary quotes |
| --- | --- |
| Preparation for aHSCT\Decision making process\Sources of information\Experiences of other people with MS | [...] I also met with several people there, who had already had stem cell transplants. And they were convinced. (pwMS 9)  Yes, a lot of it [the information] is obtained online, I'd say, and then also via Facebook groups and of course via people who have already had experience with it. (PwMS 2) |
| Preparation for the aHSCT\Decision making process\information sources\physicians | Then I sat down with my neurologist. It came up in conversation that in Switzerland stem cell transplantation is used as an alternative therapy for MS patients who have a highly active form where no other medication works. (pwMS 5) |
| Preparation for the aHSCT\Decision making process\information sources\studies and other factual information | And I really read up on it, including a lot of English texts. And then, after I had read up on it and internalized everything, I thought to myself, that this could potentially be something. (pwMS 11) |
| Preparation for aHSCT\Decision making process\Medical handling of transplantation request\Encouragement | I discussed it, but of course my neurologist could honestly say little about it (...). He supported it, but of course he couldn't say much else. (pwMS 2) |
| Preparation for aHSCT\Decision making process\ Medical handling of transplantation request \Unawareness, skepticism and rejection | (...) My neurologist, for example. I wish he had at least told me that there was this possibility. He doesn't have to recommend it to me, he doesn't have to advise me. He doesn't have to tell me to do it either. But he didn't tell me anything and I thought that was a pity. (...). (pwMS 10) |
| Preparation for aHSCT\Decision making process\Why transplant? Reasons, hopes, expectations\Failure of immunotherapies and no alternatives | And that's when I decided that stem cell therapy was the way to go, because Ocrevus doesn't work. (PwMS 4)  Because I had no other alternatives. (...) I had already tried three medications, none of which had stopped the progression. (pwMS 9) |
| Preparation for aHSCT\Decision making process\Why transplant? Reasons, hopes, expectations\Positive testimonials from other MS patients | But because of the good experience reports that were given (...), the decision was made relatively quickly to do it and to take on this huge effort and try it. (pwMS 2) |
| Preparation for aHSCT\Decision making process\Why transplant? Reasons, hopes, expectations\Hope for an improvement in symptoms | I have read that the aim of a stem cell transplant is to stop progression. But I have to say, that I have also read from one or two people that there have been some improvements. (...) And of course I secretly hoped that this would also be the case for me. (pwMS 10) |
| Preparation for aHSCT\Decision making process\Why transplant? Reasons, hopes, expectations\Hope of stopping the disease | (...) in the first step, of course (...) one hopes that the disease will first come to a standstill and that the status quo will be preserved. (pwMS 2) |
| Preparation for aHSCT\Decision making process\Why transplant? Reasons, hopes, expectations\Hope for a cure | Yes, that the whole thing works. In other words, that I'm one of those people who really get rid of the disease for life. (pwMS 3) |
| Preparation for aHSCT\Decision making process\Why transplant? Reasons, hopes, expectations\Fear of permanent and progressive disability | (...) Because, if I don't do anything, I'll die relatively quickly or, to put it bluntly, I'll vegetate in a nursing bed. (...). (pwMS 8) |
| Preparation for aHSCT\Decision making process\Why transplant? Reasons, hopes, expectations\Fear of self-reproach for not having done everything | I was 41 at the time and said: “I still have a few years ahead of me in this beautiful world. And I don't want it to be over yet.” And I don't want to blame myself in 20 years' time for not having tried everything. (pwMS 10) |
| Preparation for aHSCT\Decision making process\Why transplant? Reasons, hopes, expectations\Convincing principle of action and study data on aHSCT | You basically turn off the immune system once. And that it can build itself up again. (...) I had a functioning immune system until I was 25 (...). And then I thought maybe it could do it again. (pwMS 11) |
| Preparation for aHSCT\Decision making process\Why transplant? Reasons, hopes, expectations\Wish to be there for the family | To see my family and my child grow up. Not to go into a nursing home and not to die. That was my hope. And it was fulfilled. All good. (pwMS 6) |
| Preparation for aHSCT\Decision making process\Why transplant? Reasons, hopes, expectations\Reasons for deciding on aHSCT abroad\High level of expertise abroad | And then I chose Florence, because it's within Europe. And Dr. Saccardi in Florence also has a lot of experience with stem cell transplants for MS. (pwMS 9) |
| Preparation for aHSCT\Decision making process\Why transplant? Reasons, hopes, expectations\Reasons for deciding on aHSCT abroad\Access restrictions and rejections in Germany | And precisely because this is not yet approved as a broad therapy in Germany, but is rather difficult, the easiest option was to have it done in England. (pwMS 12) |
| Preparation for aHSCT\Decision making process\ Concerns and problems\Fear of side effects, complications and death | The main concern was dying in Moscow. That is a theoretical possibility. Of course, you think about it in advance. (pwMS 3) |
| Preparation for the aHSCT\Decision making process\ Concerns and problems\Fertility | So, I had my sperm frozen in advance for example, even though I don't have any plans to have children at the moment, it was still important to me. (pwMS 7) |
| Preparation for aHSCT\Decision making process\Concerns and problems\Therapy failure | And also, that it wouldn't work. That it wouldn't work and that I would get more disabilities. (pwMS 9) |
| Preparation for aHSCT\Decision making process\Concerns and problems\Delay in transplantation | I need to get the whole thing done as quickly as possible. And the only option available is Moscow. And in the end, that's how the stem cell transplant came about in Moscow in January 2019. (pwMS 3) |
| Preparation for the aHSCT\decision making process\Concerns and problems\child separation | So, my biggest worry was actually being separated from my children, because they are still quite small. My twin boys are 2 ½. I've never been separated from them. (pwMS 1) |
| Preparation for aHSCT\Decision making process\Concerns and problems\Lack of trust in foreign medical care | And then there was also [aHSCT] in India, but I definitely didn't want to go there. It was too risky for me and the culture there is just too different. And I had also heard of several deaths there. (pwMS 9) |
| Preparation for aHSCT\Decision making process\Concerns and problems\Lack of testimonials from other MS sufferers | The most difficult thing for me was, that I (...) had no contact person in Germany. No patient, who could really tell me what it was like and how it felt. There was simply no contact person. The international groups are all well and good. But it's always better to have someone you can communicate with in the local language. (pwMS 3) |
| Preparation for aHSCT\Decision making process\Concerns and problems\Dealing with concerns and problems\Confidence in medical expertise | So [the aHSCT] is probably already performed at a decent medical standard. (...) I saw a few videos, also about Moscow, and then I saw:  Yes, that's neat. (pwMS 10) |
| Preparation for the aHSCT\Decision making process\ Conerns and problems\Dealing with concnerns and problems\Psychological support | I prepared myself very well. I looked for a psychotherapist, who comes from research herself. Her husband is a professor of physiology. They gave me the best possible support. I did autogenic training and kept imagining the situation when I was lying there, when the stem cells, it's just like plasmapheresis, it's nothing different or anything. But I don't know, I actually prepared myself well for it. (pwMS 6) |
| Preparation for the aHSCT\Decision making process\ Concerns and problems\Dealing with concerns and problems\Faith | I also somehow believe in something (...), in something divine somewhere. I thought that something would somehow guide me through. (pwMS 6) |
| Preparation for the aHSCT\Decision making process\ Concerns and problems\Dealing with concerns and problems\Plunging into work | As I said, first I threw myself into work and everything else. And then came the phase before the transplant, when I was rather sad and hopeless. (pwMS 5) |
| Preparation for aHSCT\The rocky road to transplantation\Organizational efforts\Mostly independent search for aHSCT clinics | By writing letters, actively in letter form, asking for a change of therapy, stem cell therapy, so writing to the big clinics in Germany (...) the work involved, two years in total I would say, is enormous. Because you always have to stay on the ball. You always have to look and look and look and, above all, do a lot of paperwork. (pwMS 4) |
| Preparation for aHSCT\The rocky road to transplantation\Organizational efforts\Cancellations in Germany; fewer obstacles abroad | That was relatively uncomplicated. There is this contact person in Moscow, who always looks after foreign patients. (...) I was accepted relatively quickly, just with this waiting time before. (pwMS 3) |
| Preparation for aHSCT\The rocky road to transplantation\Organizational efforts\Postponing aHSCT due to Covid-19 | Yes, this year has simply been a year of anxious waiting. Because you've now decided to have therapy, but you can't do it because of corona. That was frustrating. (pwMS 12) |
| Preparation for aHSCT\The rocky road to transplantation\Organizational efforts\Planning the stay abroad | Yes (...) then I had another month (...) to prepare for Moscow with a visa and I had preliminary examinations done here to be on the safe side (...). You really need a month. (pwMS 8) |
| Preparation for aHSCT\The rocky road to transplantation\Financing difficulties\Coverage by the health insurance company | And then the financing was the big issue at first, because the stem cell transplant would not be carried out, if the financing was not completely clarified. Then I transferred €50,000.00 to the UKE (...). At the same time, I submitted an urgent application to the health insurance company to have the costs covered (...). So I got the costs covered (...). (pwMS 1) |
| Preparation for aHSCT\The rocky road to transplantation\Financing difficulties\Private financing (savings, crowdfunding) | As a foreigner it's not like that, [it's] difficult with the health insurance (...). Of course you have to pay for it yourself. I then started a crowdfunding campaign and raised the majority of the costs and that's how I financed it. (pwMS 12) |
| Experiencing the procedure\Positive experiences and gain of control\Care by medical staff\Psychological support | I had a psychologist at my side. Which was very important, and I was in contact with her by phone. Because I didn't get a psychologist in oncology or hematology, because I was [being cared for] neurologically. And that really helped me. (pwMS 6) |
| Experiencing the procedure\Positive experiences and gain of control\Care by medical staff\Trust in medical expertise | "Mr. pwMS 4, we're doing this, it's all normal." I knew I was with professionals. When the professionals say it's normal, you just believe them, you take note and they help you. They helped me. (pwMS 4) |
| Experience the procedure\Positive experiences and gain of control\Care by medical staff\High commitment of medical staff | The nurses are always super nice and if you ring the bell, they always come straight away and ask how you are. Even if you feel sick after chemotherapy, even just a little sick, you have to ring the bell straight away and say, I can't stand this or the nausea is coming on again and then you always get something straight away. (pwMS 1) |
| Experiencing the procedure\Positive experiences and gain of control\Care by medical staff\Much understanding and helpfulness | That the nursing staff and doctors around me were always attentive. You could express your fears and complaints in any situation. They were very sympathetic. And despite this very exciting situation, I would say, you still felt somehow safe. Of course, you also had to put yourself in their hands, because otherwise you wouldn't be able to get through it, I'll put it that way. (pwMS 5) |
| Experiencing the procedure\Positive experiences and gain of control\Treatment as time out | I thought it would be much harder than it ended up being. I thought it would be very hard to be in such a small room for a month. But in the end ... it's not a vacation, but I was able to read a lot, watch a lot of TV, have a lot of time to myself. So that wasn't so bad. (pwMS 9) |
| Experiencing the procedure\Positive experiences and gain of control\Treatment as time out\Rest | I would say that boredom was limited for me, because overall in therapy you're honestly glad, that you can lie down somewhere and have some peace and quiet. There really were days when I spent the whole day lying in bed or wherever on the couch and was happy not to have to communicate much. (pwMS 2) |
| Experiencing the procedure\Positive experiences and gain of control\Treatment as time out\Distraction through media and exercise | At some point, when I was a bit fitter, (...), I was able to do a bit of sport in the room. Light sports activity, so to speak. (pwMS 5) |
| Experiencing the procedure\Positive experiences and gain of control\Improvement of MS symptoms | I was motivated by the fact, that I noticed that my paraesthesia had already improved somewhat during the transplant and that my tingling hand had also improved somewhat, so to speak. (pwMS 5)  So, a short time later, just two or three days later, I had a bit more strength in my hand again for the first time. Whenever you get food, you always have to sign for it. (...) And then I did that on my own again for the first time. (pwMS 10) |
| Experiencing the procedure\Positive experiences and gain of control\Contact with fellow patients and relatives | And even then [during the hospitalization period], we had 20 minutes a day to meet on the sun deck in the open air. And we just chatted there (...). We were, (...), brothers in spirit. We were fighting on the same front. And of course, that bonded us. And I didn't feel lonely. (pwMS 10) |
| Experiencing the procedure\Positive experiences and gain of control\Contact with fellow patients and relatives\Exchange with family, friends and fellow patients | And you can turn to family and friends for all your emotional burdens. And that has been good. (pwMS 1) |
| Experiencing the procedure\Positive experiences and gain of control\Feeling of starting a new life | So, when I think back to the moment [of the transplant], it was very nice, very nervous. Yes, and then just the hope, that it would actually lead to a new life. (pwMS 7) |
| Experiencing the procedure\Difficulties during therapy\Emotional stress due to isolation and worries about infection | Yes, it was exciting, especially after the stem cell transplant, it's the time from day zero to day twelve, because the immune system is then very weakened. During this time, you are strictly isolated. (...) Are there any signs of inflammation? And of course, you are also very weakened, you can tell. (pwms 8) |
| Experiencing the procedure\Difficulties during therapy\Stress from lack of knowledge about aHSCT | That's the kind of stress I put on myself. Because that's just, (...), it was my mistake to deal with something like that without any background knowledge. (...) and then it was just stress. But apart from that it worked well. (PwMS 6) |
| Experiencing the procedure\difficulties during therapy\side effects of chemotherapy | I generally had fewer problems, although there were times when I was so weak that I couldn't even put my feet off the bed. (PwMS 7) |
| Post-treatment time \Successes and positive experiences | You just don't have much motivation to go out for a coffee or to a restaurant if you're preoccupied with the illness all day and always have this "I'm chronically ill, I can't get rid of this" in the back of your mind and there's no perspective in life. That's why I had impairments in every area before the transplant, both professionally and privately. After the transplant, only as a result of the chemotherapy. Currently no impairments at all. (pwMS 3) |
| Post-treatment time\Successes and positive experiences\Influence on the course of MS\Being Relapse-free | So that was also successful for me. But the symptoms you already have don't go away. And you can only do a lot of training afterwards to build up your body a bit. But at least I've now had eight years of standstill. No further relapses, no new symptoms. And that's why we're really happy. (pwMS 9) |
| Post-treatment time\Successes and positive experiences\Influence on the course of MS\Improvement in MS symptoms | After the therapy, (…) when I was discharged, I have to say that I'm feeling better and better. That's just a fact. [I take the] stick to the side [when walking] from time to time, when I have the feeling that I'm doing really well for a few meters, (...) that's actually the result I wanted to achieve, (...). (pwMS 4) |
| Post-treatment time \Successes and positive experiences\Influence on the course of MS\Progression stop | So apart from stopping the progression, which I was able to determine very quickly in my progression form. (...) I can determine this very quickly because I can say very quickly: Nothing has gotten worse. (pwMS 10) |
| Post-treatment time \Successes and positive experiences\Influence on the course of MS\Thoughts no longer revolve around MS | And it's nothing worth mentioning, let's just say that. Except that I'm happy that my everyday life is the way it is, without having to think about what symptoms I'm struggling with and how. (pwMS 5) |
| Post-treatment time \Successes and positive experiences\Gain in social and professional participation | I got a life back. And that is worth a lot. I can be there for my child. That's the best thing ever. (pwMS 6)  So now we really enjoy traveling. And I enjoy it all even more than before. I wasn't able to travel so much before (...). Then came the diagnosis and then I had other things to do than travel. I had to deal with medication and doctors. (pwMS 5) |
| Post-treatment time \Successes and positive experiences\Motivation to live healthier and more active\Changed health awareness and attitude to life | I used to like to waste my time [after work] on social networks or reading something on some news site and now I don't have time for that anymore because I spend two hours a day with the whole sports regime (...). And then I'm usually finished by 8 p.m. or so, so I can relax. And that gives me such a great feeling of satisfaction, that on the one hand, I've done something for myself again, for my body, (...), I haven't wasted any time. And that makes me happy. (pwMS 7) |
| Post-treatment time \Successes and positive experiences\Motivation to live healthier and more active\Exercise and sport | But apart from that, I've always had a very healthy diet, I've done a lot of sport my whole life and now I'm doing even more. Now I always do the maximum possible, so to speak. Both in sport and at work. (pwMS 12) |
| Post-treatment time \Successes and positive experiences\Motivation to live healthier and more active\Nutrition and dietary supplements | Then I make sure that my vitamin and mineral balance is always on top, for example vitamin D; five years ago, I was even undersupplied with vitamin D, (...) So my blood is checked every three months. I now have a value of exactly 75, so it can't happen that I would overdose. (pwMS 7) |
| Post-treatment time \Successes and positive experiences\Motivation to live healthier and more active\Stress reduction, relaxation and slowing down | [It helped me] to stop stressing, that I have to deliver this performance now. Instead, I've said that I'm happy with what I can do for now. (…). That's the kind of thing, quitting smoking, reducing stress. That's all I've done. (pwMS 6) |
| Post-treatment time \Successes and positive experiences\Motivation to live healthier and more active\Reduced alcohol consumption and smoking cessation | So, changing my lifestyle, before this diagnosis and stem cell transplantation I lived an incredibly unhealthy life. I was a heavy smoker, I was overweight, I avoided sport wherever I could, so as far as diet was concerned, I was just unhealthy, so I was really the ultimate negative example. That all changed when I was diagnosed. I suddenly stopped smoking, I ate more consciously, and I knew, at that point at the latest, that I was heading for a transplant and that I had to lose weight. (pwMS 3) |
| Post-treatment time \Successes and positive experiences\Maintaining the old lifestyle | I'm still careful about that. Apart from that, my lifestyle hasn't changed much. Well, because I did before, so I will continue to lead a healthy lifestyle. (PwMS 8) |
| Post-treatment time \Failures and difficulties\Restrictions in everyday life due to infection protection | As a form of protection, you're still not allowed to meet up with people. I hardly saw any friends for six months. I didn't go shopping; I didn't go to events. Nobody actually came into our house without a mask and gown so as not to spread germs. (pwMS 5) |
| Post-treatment time \Failures and difficulties\Physical complaints\Side effects of high-dose chemotherapy | Oh, it was very, very varied. It started with chronic diarrhea, then massive joint pain, especially in the knee joints, which lasted for weeks. (pwMS 3) |
| Post-treatment time \Failures and difficulties\Physical complaints\Worsening of MS symptoms and progression | Maybe it [the MS] has gotten a bit worse, but hopefully it's just progressing at least more slowly now than it did between 2015 and 2019 (pwMS 12). |
| Post-treatment time \Failures and difficulties\physical complaints\infections | I've had two infections so far. Once I had a sore throat and a bit of a fever, so I had about 38.6°C, but then I was given antibiotics again and two weeks ago my children gave me a bit of a cough again, but it wasn't bad at all. (pwMS 1) |
| Post-treatment time \Failures and difficulties\Psychological-emotional stress\Mental overload, sleep disorders and depression | I had a depression following the transplant. I think I've already mentioned that. I'm very concerned, that my psyche is doing well. (pwMS 6) |
| Post-treatment time \Failures and difficulties\Psychological-emotional stress\Fear of infection | Yes, that's one of those things, because just the other day I was thinking: "Should I go barefoot in the grass or is it still too early?" (...) because I think to myself, it's probably not a problem, but always in the back of my mind, it could be a bit of a problem. (pwMS 7) |
| Post-treatment time \Failures and difficulties\Psychological-emotional stress\Isolation and loneliness | So, the biggest problem after the stem cell transplant was the loneliness, because I was really careful to isolate myself from the others so that I didn't catch any infections. And that was probably the hardest hurdle. (pwMS 7) |
| Post-treatment time \Failures and difficulties\Psychological-emotional stress\Fear, that the effects of the transplants will not last | But of course, (...) mentally (...) you're always wavering: did it work; do I notice something new; is this a new symptom; am I having another relapse. That also took several years. Because with every cold I have or every time I get a fever, I get the old symptoms again. (pwMS 9) |
| Post-treatment time \Failures and difficulties\Coping with problems\Medical measures\Infection prevention | So, in my case, it actually overlapped with the Corona period. And then, of course, you have to be double careful not to get infected with the coronavirus, because your immune system of course is weakened. Thankfully, I've also had the opportunity to stay at home until today or to protect myself as far as possible. (pwMS 2) |
| Post-treatment time \Failures and difficulties\Coping with problems\Medical measures\Physiotherapy/exercise | Yes, afterwards I went straight to follow-up treatment, so I wasn't at home at all, because I couldn't cope any more. And I said I couldn't cope at home. And then I trained like crazy for weeks. (pwMS 6) |
| Post-treatment time \Failures and difficulties\Coping with problems\Medical measures\Medical monitoring and treatment | That was the phase afterwards, but I have an oncologist in private practice here, who checks my blood work and it's always been great, I have to say, so everyone is in good spirits. (pwMS 1) |
| Post-treatment time \Failures and difficulties\Coping with problems\Medical measures\Rehabilitation measures | And I'm now also trying to go to rehab in December, for example. I didn't want to do it before, because I felt so weak, I thought, if you're so weak, what do you want to train for, you won't be able to do it. (pwMS 8) |
| Post-treatment time \Failures and difficulties\Coping with problems\Social measures\Help from family and friends | I have to say that what helped me a lot during the transplant was my stable environment. The fact that I always had someone around me, so to speak, who looked after me, who took care of everything, because I also had to deal with all the official stuff, keeping my salary going and everything. Sometimes you're not even able to do that. And this stable environment helped me a lot. (pwMS 5) |
| Post-treatment time \Failures and difficulties\Coping with problems after\Social measures\State assistance | But I also had a lot of help, I must say. I also got household help through my health insurance. That was really worth its weight in gold. And I just tried to recover well. (pwMS 1) |
| Post-treatment time \Failures and difficulties\Coping with problems\Social measures\Care service | That's very, very, very important and of course, I also need support from my father's care service, who can still do one or two things for me at the moment and so on. I definitely need that. (pwMS 10) |
| Post-treatment time \Failures and difficulties\Coping with problems\Coping strategies\Stamina | And apart from that, I always, always, always tried to fight as much as I could. (...) And that's the thing, you can never give up and sometimes you have to lie in the mud and wallow in it until you can get up again. And if you have a certain fighting spirit, I think, then you can live with it somehow. (pwMS 11) |
| Post-treatment time \Failures and difficulties\Coping with problems\Coping strategies\Relativizing long-term risks | I don't think about that [the long-term risks]. So, if something happens, then it happens, but hopefully not. (pwMS 8)  So, there are long-term risks with all medications for MS. The risk of developing cancer is also increased with Tysabri or Ocrevus. So that didn't particularly influence my decision. (pwMS 1) |
| Post-treatment time \Failures and difficulties\Coping with problems\Coping strategies\Rest | Of course you take it easy, and this taking it easy doesn't make it any easier in terms of mobility. And of course you have to fight against it. (pwMS 2) |
| Post-treatment time \Failures and difficulties\Coping with problems\Coping strategies\Positive reassessment | But I think it has changed my attitude a bit. I'm much more grateful for everything. I'm now really, I can be extremely happy about the smallest things. (...) And I think I'm a bit more positive about life again. Because before I was so close to despair and now, I actually have a lot of positivity in me. (pwMS 11) |
| Post-treatment time \Aftercare\Remaining in contact with transplant site | But Dr. Fedorenko also gives every patient his contact details, WhatsApp and e-mail. And I've written to him twice (...) So of course it's also very practical that (...) I can send him e-mails and WhatsApp messages with questions. (pwMS 7) |
| Post-treatment time \Aftercare\Self-organization of aftercare | I now think, that the time after the hospital stay is more stressful for me, having to see who is looking after me and when do I go to the doctor and that is more the point. (pwMS 8) |
| Post-treatment time \Recommendations to people seeking aHSCT\Consideration of aHSCT at an earlier stage | Do it [the transplant] as soon as possible and think about the future. The whole thing [the MS] won't get any better. (pwMS 3) |
| Post-treatment time \Recommendations to people seeking aHSCT \Early planning of organization and financing | It's not just the transplant itself, but the whole thing also comes with a bunch of problems, so to speak. In terms of work, financially. You might have to discuss with the authorities whether you can get a severely disabled person's pass, so that you can continue to have your salary paid by the health insurance company, (...) so to speak. All these documents and applications are things you didn't even think about before. (pwMS 5) |
| Post-treatment time \Recommendations to people seeking aHSCT \Contact with T-specialists facilitates access and organization | Then you should look for a doctor who is not negative about it. So preferably some doctors who have possibly already transplanted themselves or have had patients who have already been transplanted, and who are familiar with it and don't have a negative attitude towards it. (pwMS 11) |
| Post-treatment time \Recommendations to people seeking aHSCT \Transplantation at specialized center abroad | Incidentally, if you have the money, I wouldn't go for Germany, but rather Mexico or Moscow, because they simply have much, much more experience than Germany now in 2022. (pwMS 10) |
| Post-treatment time \Recommendations to people seeking aHSCT\Cost/benefit assessment | And you also have to be aware that it is not a cure. It's not a miracle cure, it's a therapy option, an alternative therapy that you have to choose. You first have to think about whether you really want to do it or not. But then: what is the alternative? If the medication doesn't work. (pwMS 9) |
| Post-treatment time \Recommendations to people seeking aHSCT\Obtain factual information and experiences from others | And also talk to lots of different people who have already had a transplant. And especially with people who have been transplanted for a long time. Because I've also heard about a lot of people who didn't really succeed after five years. (pwMS 9)  You should definitely inform yourself beforehand. If you’re relatively good in English, you should read through all the studies, which are very interesting and answer all your questions. (pwMS 11) |
